# Generalized Odontodysplasia: A Case Report of the Oldest Reported Patient

**DOI:** 10.1155/crid/5602411

**Published:** 2025-07-14

**Authors:** Freddy Andrés Vivero-Alcívar, Lilibeth Stephania Escoto-Vásquez, Oscar Rohel Hernández-Ortega, Raymundo Ramírez-Lugo, Adriana Molotla-Fragoso

**Affiliations:** ^1^Department of Oral and Maxillofacial Surgery, Postgraduate and Research Division, Dental School, National Autonomous University of Mexico, Mexico City, Mexico; ^2^Medical, Dental and Health Sciences Program, Postgraduate and Research Division, Dental School, National Autonomous University of Mexico, Mexico City, Mexico; ^3^Department of Oral Medicine and Pathology, Postgraduate and Research Division, Dental School, National Autonomous University of Mexico, Mexico City, Mexico

**Keywords:** dental enamel hypoplasia, dentin dysplasia, ghost teeth, odontodysplasia, teeth abnormalities

## Abstract

Odontodysplasia is a dental anomaly that affects the maturation and formation of teeth, resulting in hypoplasia and hypocalcification of both enamel and dentin. It can affect one or several quadrants of the dentition, although generalized involvement is extremely rare. The exact cause is unclear, but trauma, infection, and nutritional or metabolic deficiencies have been suggested as possible contributing factors. Diagnosis requires a combination of clinical and radiological findings. Clinically, it presents as small teeth with yellow or brown discoloration, and it can affect both the primary and permanent dentition. Radiographically, there is reduced radiodensity, giving rise to a blurred or “ghost tooth” appearance. Histologically, these teeth show aprismatic enamel, interglobular dentin, and the presence of enamel-like calcifications known as enameloid conglomerates. Treatment depends on the extent of tooth involvement and the patient's age. This paper reports the case of a 31-year-old woman who presented with this rare anomaly in its generalized form, making her the oldest patient with this diagnosis reported in the literature.

## 1. Introduction

Odontodysplasia is a dental anomaly that affects tooth maturation and formation [1]. Hitchin first reported the condition in 1934, but the term “Odontodysplasia” was introduced later by Zegarelli et al. in 1963 [2]. Regional odontodysplasia occurs when the condition affects one or more teeth in the same quadrant. When all four quadrants are involved, it is known as generalized odontodysplasia, a condition that is extremely rare [3]. To date, only 10 cases have been reported [4]. The etiology of odontodysplasia remains uncertain. However, different theories suggest that local and genetic factors may be related to its development [1, 2].

Diagnosis requires the correlation of clinical and radiographic characteristics. Clinically, affected teeth are typically small, with altered morphology. The enamel and dentin are hypoplastic and hypocalcified, resulting in yellow or brown discoloration and a rough surface. Other signs may include dental mobility, early loss of primary teeth, delayed tooth eruption, localized pain, and fistulas. The maxilla is affected more frequently than the mandible. While there appears to be no overall gender predilection, some studies have suggested a higher incidence in females. The age at diagnosis usually ranges from 1 to 23 years and can involve both primary and permanent dentition [2, 4].

Radiographically, characteristic findings include wide pulp chambers with open apices and a blurred demarcation at the dentin-enamel junction. Due to defects primarily in the formation and maturation of enamel and dentin, reduced radiodensity is observed, resulting in the teeth having a blurred or “ghost tooth” appearance [2, 4]. Histologically, both dentin and enamel appear hypomineralized (deficient in minerals) and show marked disorganization. The enamel lacks the normal rod-like structures called prisms and appears “aprismatic.” Additionally, irregular tubular dentin and areas of interglobular dentin (areas with less mineralization between the tubules) are present, along with pulp chambers containing calcifications [1, 4]. Cementum may be absent or present in smaller amounts in some areas and may have a globular appearance [5]. The follicular tissue surrounding the developing tooth crown can also be affected in odontodysplasia. This tissue may be enlarged and often contains focal accumulations of basophilic (blue staining) enamel-like calcifications, called enameloid conglomerates [5]. Treatment should be individualized and require a multidisciplinary approach [2], depending on the degree of tooth involvement and the patient's age. Treatment options are individualized based on the degree of tooth involvement and the patient's age, and may include conservative restoration, pulpotomy/root canal therapy, tooth extraction, and partial acrylic dentures. In patients of appropriate age, some studies suggest the placement of implants with fixed restorations [3, 4]. This report describes the case of a patient diagnosed with generalized odontodysplasia, who is the oldest patient documented in the literature to date. It also highlights the importance of diagnosing the condition through the correlation of clinical, radiographic, and pathological findings, as well as considering appropriate treatment options.

## 2. Case Report

**Figure 1:**
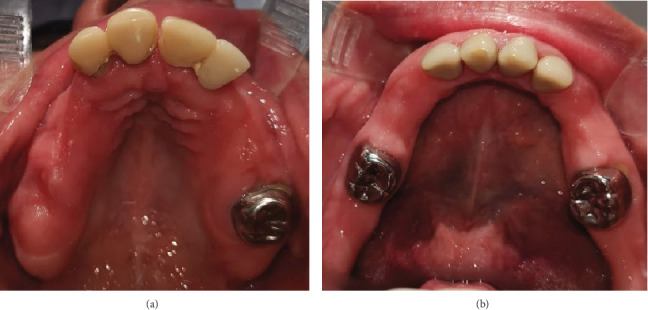
Intraoral photography. (a) Upper jaw where Teeth 13, 14, 15, 16, 17, 23, 24, and 25 are absent. (b) Inferior jaw with canines and premolars absent on both sides. Multiple irregularities in the alveolar bone.

A 31-year-old female patient with no significant medical history relevant to her current condition was referred to the Oral and Maxillofacial Surgery Department at the Postgraduate and Research Division, Dental School, UNAM (Mexico City, Mexico) due to complaints of “multiple dental retention”. The patient's primary concern was the absence of many teeth in her mouth, stating that she would like fixed dentures because the teeth that had erupted were crumbling. Upon physical examination, Class III malocclusion was noted. The intraoral exam revealed that both the upper and lower central and lateral incisors had porcelain-fused-to-metal crowns, while the upper and lower right first molars had only metal crowns. The clinical examination also showed the absence of several teeth in both the upper and lower jaws (Figure 1).

**Figure 2:**
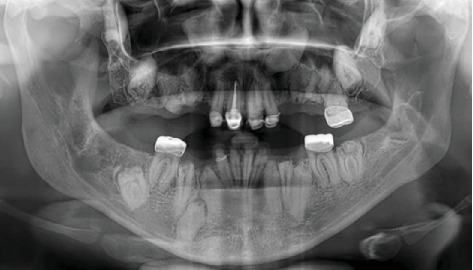
Panoramic radiography. Multiple impacted teeth and “ghost teeth” present.

Radiographic examination revealed multiple impacted teeth in both the maxilla and mandible. A distinctive feature of these impacted teeth was the lack of differentiation between the radiopacity of the enamel and dentin, creating a “ghost tooth” appearance. This finding raised suspicion for generalized odontodysplasia (Figure 2).

**Figure 3:**
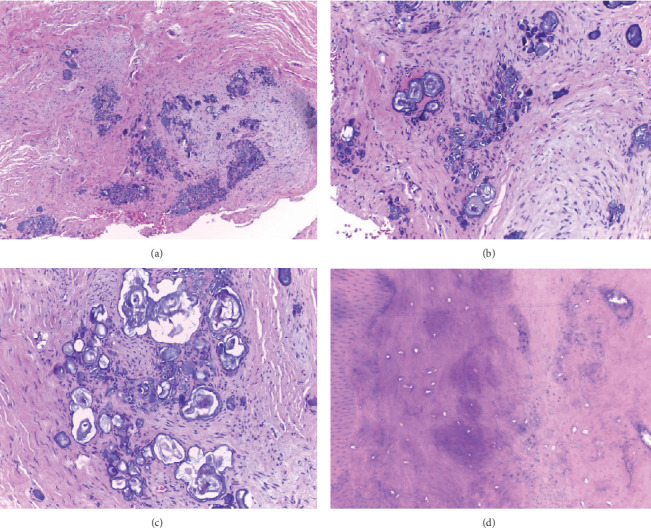
H&E photomicrographs. (a) 100x—fragment of dense irregular connective tissue with sparse distribution of cells and multiple basophilic calcifications arranged in focal areas. (b) 100x—concentric calcifications of varying sizes interspersed with connective tissue resembling the dental papilla. (c) 200x—conglomerates of tissue similar to enamel. (d) 200x—amorphous matrix resembling osteodentin, with varying degrees of mineralization.

Given these clinical and radiographic findings, a decision was made to extract the lower right third molar, which had soft tissue adhesions. The extracted tooth, along with the included soft tissue adhesions, was placed in 10% formalin and sent to the Department of Oral Medicine and Pathology at DEPeI-UNAM (Mexico City, Mexico) for histopathological analysis. The histopathological examination revealed irregular dense connective tissue, conglomerates of enamel-like tissue with basophilic calcifications of various sizes, and areas with eosinophilic amorphous matrices, all consistent with dysplastic dentin. Mature bone lamellae with signs of resorption were observed, along with islands of oval-shaped cells, consistent with epithelial rests. These findings confirmed the diagnosis of odontodysplasia (Figure 3).

**Figure 4:**
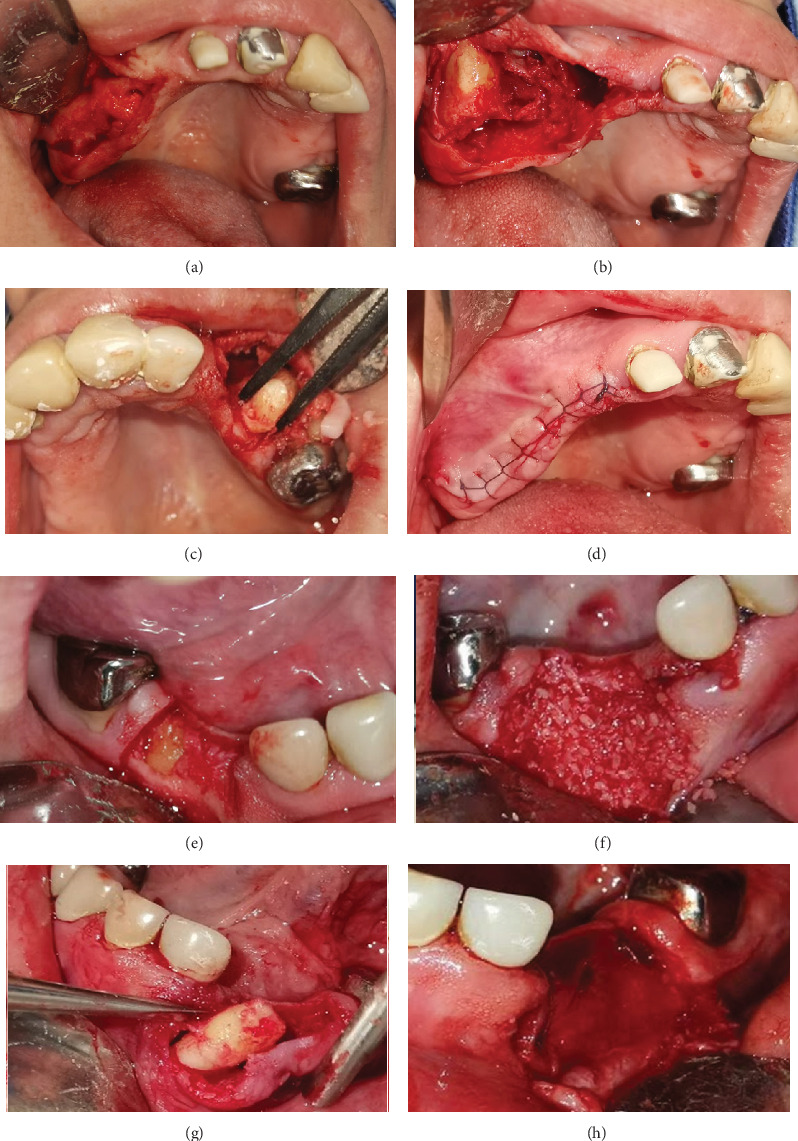
Surgical extraction of impacted teeth. (a) Flap design. (b, c) Tooth exposure with ostectomy. (d) Flap repositioning and suturing. (e) Flap design on the right side of the mandible. (f) One graft placed in the extraction socket. (g) Flap design on the left side of the mandible. (h) Collagen membrane.

**Figure 5:**
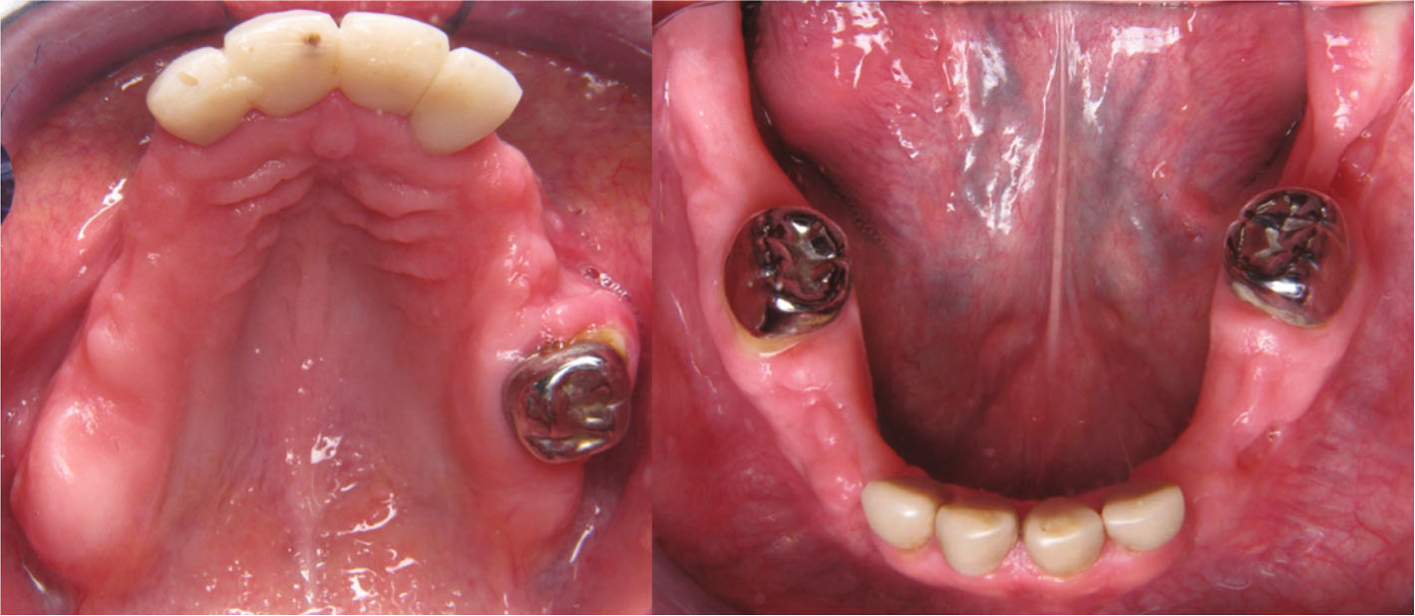
Postoperative control after a week.

After confirmation of the diagnosis, treatment was planned, and extractions were performed under local anesthesia. Impacted teeth were extracted, and alveolar preservation was carried out in the mandible (Figure 4). A week later, the patient returned for postoperative control (Figure 5), where good healing of the soft tissues was confirmed, and the sutures were removed.

**Figure 6:**
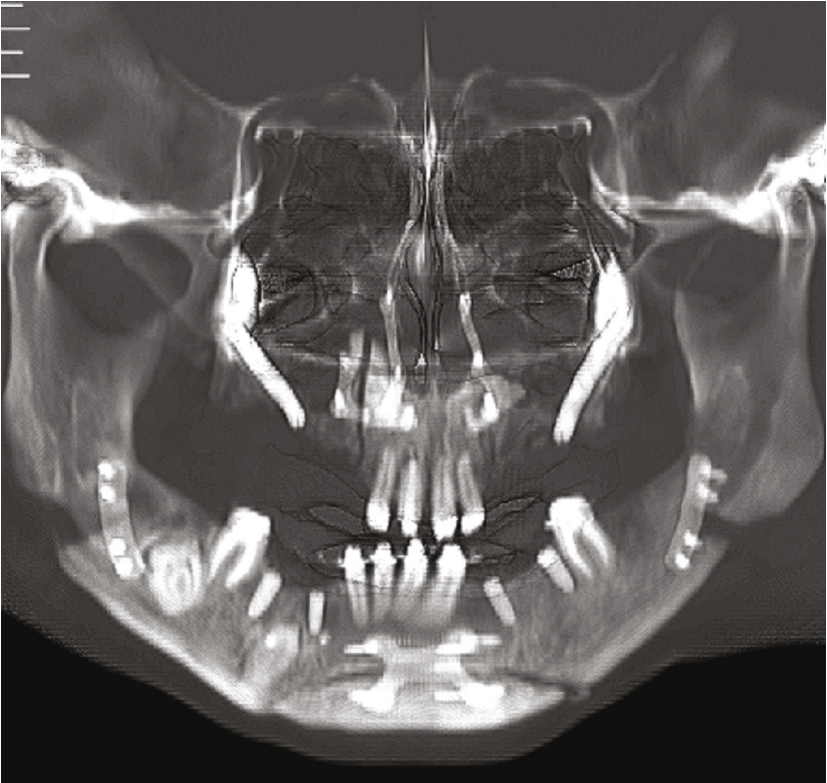
Five-month follow-up tomography panoramic reconstruction.

To facilitate future implant placement, a bovine bone graft and a collagen membrane were used to fill the bone defects in the mandible. Teeth 26, 36, and 46, along with the upper and lower central and lateral incisors, were preserved to maintain the vertical dimension. However, the long-term treatment plan involves extracting the upper central incisors to make room for a complete upper denture, which will help correct the patient's maxillary hypoplasia (Figure 6).

## 3. Discussion

Odontodysplasia is a rare developmental anomaly that affects dental tissues derived from both mesodermal and ectodermal origins [2] The condition may involve a specific area or quadrant in one or both dentitions, typically in the maxilla or mandible, though it rarely affects both jaws entirely [6]. Hitchin described this condition in 1934, although McCall and Wald first reported it in 1947, referring to it as “arrested dental development” [7]. In 1954, Rushton introduced the term “shell tooth” to describe the characteristic radiological features, while Zegarelli coined the term “odontodysplasia” in 1963. Later, in 1970, Pindborg added the term “regional” due to the condition's tendency to affect specific quadrants of the dentition [4, 6, 7]. The prevalence of odontodysplasia is extremely low, estimated at less than 1 per 1,000,000 individuals. Two studies highlight this rarity: Alotaibi et al. analyzed 161 cases reported between 1953 and 2017, and Nijakowski et al. reviewed 180 cases up to September 2021 [2]. Interestingly, both studies suggest a possible gender predisposition, with a female-to-male ratio of 1.4:1 [4].  Table 1 summarizes other epidemiological features of odontodysplasia.

Due to limitations in data collection, these prevalence figures should be considered estimates, as they are based solely on case reports published in English. As a result, it is uncertain whether these distributions accurately represent the broader population affected by odontodysplasia [8]. As shown in Table 1, generalized odontodysplasia accounts for less than 5.5% of reported cases. According to Nijakowski et al. [4], only eight cases had been documented up to the time of their study.

The etiology of this dental anomaly remains unknown [9, 10]. Several factors have been proposed as potential contributors, including trauma, local infections, medications taken during pregnancy, blood supply issues to the teeth, Rh incompatibility, radiation therapy, neurological trauma, fever, metabolic and nutritional disorders, and vitamin deficiencies [2, 9, 11, 12]. Initially, biological inheritance was not considered a significant factor due to the absence of familial cases [11]. However, a 2019 study by Koskinen et al. challenged this view [10]. They reported the case of a 6-year-old patient with regional odontodysplasia and a family history of oligodontia. In this study, genes associated with oligodontia (MSX1, PAX9, AXIN2, and WNT10A) were analyzed in both the patient and her relatives with oligodontia, revealing the same mutation in the initial codon of the PAX9 gene [10]. While this finding suggests a potential genetic link, further research is needed to confirm this association. Our patient had no family history of odontodysplasia nor oligodontia.

Odontodysplasia has been associated with a greater or lesser degree of hypoplasia of the affected side of the face, ipsilateral and contralateral vascular nevi, bilateral epicanthus, hypertrophic labial frenum, hearing loss, hydrocephalus, orbital cyst, and nasopalatine duct cyst [13]. Additionally, both regional and generalized odontodysplasia have been linked to certain syndromic conditions, including epidermal nevus syndrome and, more recently, PHACE (posterior fossa malformation, hemangioma, arterial anomalies, coarctation of the aorta/cardiac defects, and eye abnormalities) syndrome [14–16].

Epidermal nevus syndrome, also known as “linear sebaceous nevus syndrome,” is defined by a combination of nevoid skin alterations, epileptic seizures, and psychomotor retardation [14].

The syndrome's most prominent feature is an extensive congenital linear epidermal nevus, typically located on the face and scalp. In some cases, dental manifestations resemble odontodysplasia, suggesting that odontodysplasia may share a similar etiology and could represent one end of a spectrum of this condition [13–15]. PHACE syndrome, recently associated with odontodysplasia, was first described in 1996. The acronym PHACE was later updated to include sternal malformations [16]. The diagnostic criteria for PHACE syndrome include infantile hemangioma, which typically affects the face, scalp, or cervical region. It has been hypothesized that hemangioma could contribute to an irregular blood supply during tooth bud development, leading to subsequent abnormal tooth development. This suggests that disturbances in the blood supply may play a significant role in the etiology of odontodysplasia [16, 17]. Clinically, several features have been reported in patients with this condition [4], including delayed tooth eruption, mild facial asymmetry, yellowish-brown crowns, and alterations in dental morphology. These changes may lead to complications such as gingival hyperplasia, abscesses, fistulas, and pain in the affected areas [11, 18, 19].

It is important to note that not all these clinical signs are present in every case. However, delayed tooth eruption is a key indicator that often prompts radiographic evaluation. Such examinations may reveal the characteristic “ghost teeth” appearance typically associated with odontodysplasia [8, 18]. In the present case, the patient exhibited multiple missing teeth and irregularities in the alveolar ridge. Initially, these findings could have been confused with prior tooth extractions. However, a thorough evaluation, including clinical examination, medical history, and radiographic analysis, led to a preliminary diagnosis of generalized odontodysplasia.

A key radiographic feature of odontodysplasia is the presence of “ghost teeth,” which can be seen in up to 92.2% of the affected teeth [4]. These “ghost teeth” appear due to alterations in the mineralization process, resulting in a significant reduction in the radiopacity of both the enamel and dentin. This makes it difficult to distinguish between the two, leading to a widening of the pulp chamber and sometimes the presence of calcifications within it [4, 5].

Histologically, all dental germ structures are affected. The enamel shows variations in thickness and arrangement of the prisms, leading to an irregular appearance with areas of hypoplasia and hypocalcification, while enamel near the amelocementary junction may appear more normal [5].

The dentin shows a reduced number of dentinal tubules, with large amounts of amorphous material present, which corresponds to interglobular and globular dentin. The most notable feature is the presence of osteodentin, an amorphous material that, in some cases, shows signs of bone metaplasia. Previous studies have shown that this matrix is rich in glycosaminoglycans and reticulin, indicating that it is immature dentin [5, 20]. Cementum may be absent or present in smaller quantities in certain areas and is often observed with a globular appearance. In the pulp, calcifications can be seen, displaying tubular or laminar structures [5, 20]. In the follicular tissue surrounding the crown, islands of odontogenic epithelium and basophilic calcifications of enamel-like tissue, known as enameloid conglomerates, are commonly observed. However, this finding is not pathognomonic of odontodysplasia, as it can also be seen in other conditions affecting enamel structure, such as amelogenesis imperfecta [5, 20]. The treatment of odontodysplasia varies depending on the severity of the condition [4]. In some cases, conservative approaches such as restorations or root canal treatments may be effective [19]. However, tooth extraction is the most common intervention, performed in 78.6% of patients [4]. This high rate of extraction is partly due to the failure of conservative treatments over time, which often necessitate extractions in the long term [4, 6]. For younger patients, whenever feasible, dentists prioritize conservative treatments that help preserve the teeth [7, 8]. The goal is to maintain the integrity of the teeth and promote healthy jawbone development, which will support future implant-supported dentures. As patients age, aesthetic considerations become increasingly important in treatment planning [11].

## 4. Conclusions

The various characteristics of generalized odontodysplasia can make diagnosis challenging, especially if the clinician is unfamiliar with the condition. One important feature to consider during the clinical examination is the dental inclusion, which, when correlated with radiographic findings, can help guide the diagnosis if “ghost teeth” is present. However, a histopathological study, whenever possible, is essential to confirm dysplasia in multiple dental tissues, allowing for differentiation from other conditions that affect dental structures. Treatment for these patients varies based on the severity of the odontodysplasia, as well as the patient's age, needs, and expectations, ranging from conservative management to more complex multidisciplinary interventions.

## Figures and Tables

**Table 1:** Epidemiologic features of regional odontodysplasia.

**Feature**		**Nijakowski et al. [** 4 **]**	**Alotaibi et al. [** 2 **]**
Sex	Men	49.4%	42.2%
	Women	50.6%	57.8%

Dentition	Primary	5%	19.3%
	Permanent	28.9%	34.2%
	Mixed	66.1%	46.6%

Jaw	Maxillary	61.1%	55.9%
	Mandible	30%	33.5%
	Both	8.9%	10.6%

Location	Right maxilla	21.7%	24.8%
	Left maxilla	25%	25.5%
	Right mandible	11.7%	13%
	Left mandible	10%	11.2%
	Bilateral maxillary	14.4%	4.3%
	Bilateral mandible	8.3%	9.3%
	Generalized	5.5%	4.3%

	One tooth	N/A^a^	1.2%

*Note:* Adapted from Nijakowski et al. [4] and Alotaibi et al. [2].

^a^Nijakowski et al. [4] did not report cases where patients only had one tooth affected with odontodysplasia.

## Data Availability

The data that support the findings of this study are available from the corresponding author upon reasonable request.
